# HIST2H2BF Potentiates the Propagation of Cancer Stem Cells *via* Notch Signaling to Promote Malignancy and Liver Metastasis in Colorectal Carcinoma

**DOI:** 10.3389/fonc.2021.677646

**Published:** 2021-08-12

**Authors:** Lei Qiu, Xiuwei Yang, Jingyu Wu, Changzhi Huang, Yongchang Miao, Zan Fu

**Affiliations:** ^1^Department of General Surgery, The First Affiliated Hospital of Nanjing Medical University, Nanjing, China; ^2^Department of General Surgery, The Second People’s Hospital of Lianyungang, Lianyungang Tumor Hospital, Lianyungang Hospital Affiliated to Bengbu Medical University, Lianyungang, China

**Keywords:** HIST2H2BF, DNA methylation, cancer stem cells, liver metastasis, Notch pathway

## Abstract

**Background:**

Growing evidence demonstrates that the initiation and progression of colorectal carcinoma (CRC) is related to the presence of cancer stem cells (CSCs). However, the mechanism through which the stem cell features of CRC cells are maintained is poorly understood. In this study, we identified the oncogenic histone cluster 2 H2B family member F (HIST2H2BF) and aimed to investigate the function of upregulated HIST2H2BF expression in maintaining the stem cell features of CRC cells, which accelerate the progression of CRC.

**Methods:**

HIST2H2BF expression was quantified using real-time polymerase chain reaction, immunohistochemistry, and western blotting. The correlation between CpG island methylation status and HIST2H2BF re-expression was assessed through bisulfite sequencing polymerase chain reaction, methylation-specific polymerase chain reaction, and 5-Aza-dC treatment. Functional assays were performed on CRC cells and mice to investigate the HIST2H2BF-induced stem cell-like and cancer properties of CRC. Using the Notch pathway inhibitor FLI-06, the regulatory effect of HIST2H2BF on downstream Notch signaling was confirmed.

**Results:**

HIST2H2BF was highly expressed in CRC tissues and cell lines. The reactivation of HIST2H2BF in CRC stems at least in part from the hypomethylated CpG islands. CRC patients with high HIST2H2BF expression have poor survival outcomes. Functional studies have shown that HIST2H2BF promotes CSC phenotype, malignancy, and liver metastasis through the activation of Notch signaling in CRC. Blockage of the Notch pathway reduced the stem cell-like and cancer properties.

**Conclusion:**

Our study suggests that HIST2H2BF upregulation enhances the CSC phenotype, malignancy, and liver metastasis through the activation of Notch signaling in CRC. These results identified a new perspective on the mechanism by which the stem cell features of CRC cells are maintained and highlighted the potential novel therapeutic targets for CRC.

## Introduction

Colorectal carcinoma (CRC) is one of the most common types of cancer in the world (1–3). Despite the advances in CRC treatment, patients experience a high rate of recurrence and resistance to chemotherapy (3–5). Consequently, novel interventions for CRC are urgently needed to enlighten the advances in novel treatment strategies and improve the prognosis of patients with CRC.

Previous studies have demonstrated that epigenetic modifications are involved in the development and progression of CRC, altering the gene expression without changing the original DNA sequence (6). DNA methylation, which is a common epigenetic modification, occurs mainly in promoter regions and often drives gene silencing (7, 8). The methylation modifications of specific genes are correlated with the progression of CRC (9). However, the precise mechanism still needs to be elucidated.

Compelling evidence has shown that CSCs, also considered as tumor-initiating cells (TICs), are the primary cells responsible for the seeding and colonization of distant metastases (10, 11). CSCs possess the capacity for self-renewal, heterogeneous lineage differentiation, clonal tumor initiation, and distant repopulation potential. The CSC hypothesis indicates that similar to normal colorectal tissues, CRC cells are organized hierarchically and depend on CSCs for population maintenance (12, 13). Accumulating evidence has demonstrated that CSCs play an important role in the poor prognosis and relapse of cancers, including CRC (14, 15). Thus, the development of an alternative strategy for targeting CSCs is highly desirable. Nevertheless, the underlying mechanisms of CSC emergence and expansion in CRC remain unclear.

To our knowledge, only a few studies have reported the association between HIST2H2BF and cancer development. J Castillo et al. claimed that proteomic analysis of the exosome “surfaceome” demonstrated a series of pancreatic ductal adenocarcinoma-specific biomarker candidates: HIST2H2BE, HIST2H2BF, EPCAM, CLDN4, LGALS3BP, and CD151 (16). Zeng et al. reported that HIST2H2BF could act as a new biomarker for the prognosis of lung cancer (17). However, it remains unknown whether HIST2H2BF plays a vital role in CRC. The clinicopathological significance of HIST2H2BF in the development of CRC still needs to be investigated, and no study has uncovered the functional role of HIST2H2BF in the development of CRC.

Here, we aimed to identify the correlation between CpG island methylation status and HIST2H2BF re-expression and the oncogenic role of HIST2H2BF in human colorectal CSCs. The biological, mechanistic, and clinical implications of our study clarified the mechanisms of CRC malignancy and liver metastasis and provided a novel prognostic biomarker and therapeutic target for patients with CRC.

## Materials and Methods

### CRC Tissues and Cell Culture

A total of 100 paired CRC tissues and their corresponding adjacent normal tissues were obtained from CRC patients who underwent primary resection without preoperative chemoradiotherapy at Jiangsu Province Hospital (Nanjing, China) between 2011 and 2016. All patients provided a written informed consent. The CRC cell lines (LOVO, HCT116, DLD-1, HT29, and SW480) and the human normal colon epithelial cell line NCM460 were derived from the Chinese Academy of Science (China) and cultured as previously reported (18, 19).

### Lentivirus and Reagents

To upregulate and downregulate the expression of HIST2H2BF, commercially available lentiviral vectors encoding HIST2H2BF and short hairpin RNAs targeting HIST2H2BF were synthesized by GeneChem (Shanghai, China). The empty lentiviral construct served as a negative control (vector *versus* overexpression; control *versus* knockdown). These constructs were verified by DNA sequencing before being used to overexpress or knockdown HIST2H2BF in CRC cells. The infected cell lines were harvested after selection with 5 μg/ml of puromycin for 10 days.

### Cell Counting Kit-8 Assays

In line with the manufacturers’ protocols, Cell Counting Kit-8 (CCK-8) (Dojindo, Japan) was used to analyze the proliferation of CRC cells. Briefly, the CRC cells (500 cells/100 μL) were seeded, and 10 μL of Cell Counting Kit-8 solution was added at the same time of each day. After incubating for 2 h in an incubator, the absorbance (450 nm) was measured.

### Clonogenic Assay

Stable CRC cells (1 × 10^3^) were cultured in a 6-well plate in Dulbecco’s Modified Eagle Medium for 2 weeks. Proliferating colonies were stained with crystal violet (Beyotime, Shanghai, China), and colonies consisting of 50 cells or more were counted and photographed for statistical analysis. All procedures were performed in triplicate.

### Apoptosis Assay

Stable CRC cells (3 × 10^3^) cells were treated with or without various concentrations of 5-fluorouracil (5-FU) (0, 2, 4, and 8 μg/ml) and cisplatin (0, 25, 50, and 100 μg/ml) for 2 days. The cells were then collected and stained with fluorescein isothiocyanate-conjugated annexin V and propidium iodide. The harvested cells were detected using a flow cytometer on a BD FACSCanto II, and the data were analyzed using the FlowJo software.

### Migration and Invasion Assays

As described previously (20–22), transwell inserts (Corning Inc., Corning, NY, USA) with or without Matrigel (BD Biosciences, San Diego, CA, USA) were used to evaluate cell invasion and migration, respectively. For cell invasion assay, the upper chambers were coated with Matrigel. A total of 2 × 10^4^ cells were seeded into the upper chamber filled with 200 μl of serum-free medium. Then, the lower chambers were added with 600 μl of Dulbecco’s Modified Eagle Medium containing 10% fetal bovine serum. After 24 h of incubation, the invaded cells were fixed with 4% paraformaldehyde, stained with 1% crystal violet, and photographed under a microscope. Cell migration assay was carried out in a similar manner without coating the upper chambers with Matrigel.

### Quantitative Reverse Transcription-Polymerase Chain Reaction

Total RNA was extracted from CRC tissues and cell lines with a TRIzol reagent (Invitrogen). cDNA synthesis was carried out using PrimeScript RT Master Mix (TaKaRa, Dalian, China), while reverse transcription-polymerase chain reaction (PCR) was performed using TB Green Premix Ex Taq (TaKaRa) on the 7900HT Fast Real‐Time PCR System (Applied Biosystems, Foster City, CA, USA; Thermo Fisher Scientific). β-actin was used as the internal control. The primer sequences were as follows: HIST2H2BF: forward: 5′-TCCAAAAAGGCTGTTACGAAAG-3′, reverse: 5′-GTTGACGAAGGAGTTCATGATG-3′; CD133: forward: 5′-CACTACCAAGGACAAGGCGT-3′, reverse: 5′-TCCAACGCCTCTTTGGTCTC-3′; CD44: forward: 5′- CACACCCTCCCCTCATTCAC-3′, reverse: 5′-CAGCTGTCCCTGTTGTCGAA-3′; ABCG2: forward: 5′- GCATCGATCTCTCACCCTGG-3′, reverse: 5′-ATTGCTGCTGTGCAACAGTG-3′; ALDH1: forward: 5′-TGCCGGGAAAAGCAATCTGA-3′, reverse: 5′-AGCATTGTCCAAGTCGGCAT-3′; Nanog: forward: 5′-GGGCACTTACGTGCATTGT-3′, reverse: 5′- GCAGGCACAAGATGGGAAAAG-3′; Bmi-1: forward: 5′-CGCTTGGCTCGCATTCATT-3′, reverse: 5′-TTGCTGGTCTCCAGGTAACG-3′; Oct-4: forward: 5′-CCGTATGAGTTCTGTGGGGG-3′, reverse: 5′-CCAGCTTCTCCTTCTCCAGC-3′; and β-actin: forward: 5′-TGACGTGGACATCCGCAAAG-3′, reverse: 5′-CTGGAAGGTGGACAGCGAGG-3′.

### Western Blotting

CRC tissues and cell lines were collected and lysed in radioimmunoprecipitation assay buffer with PMSF (Beyotime, Shanghai, China). Protein samples were separated by sodium dodecyl sulphate–polyacrylamide gel electrophoresis and transferred in the polyvinylidene difluoride membranes (Millipore). Then, 5% non-fat milk was used to block the membranes for 2 h. Subsequently, the membranes were incubated with primary antibodies at 4°C overnight and incubated with the corresponding horseradish peroxidase-conjugated secondary antibody. Each band was visualized by enhanced chemiluminescence reagents (Yeasen, Shanghai, China). The following antibodies were used for Western blotting: HIST2H2BF (Thermo Fisher Scientific, USA, 1:1000), NICD (CST, Beverly, MA, USA, 1:1000), Hes1 (CST, 1:1000), Hey1 (Abcam, 1:1000), CD133 (Abcam, 1:1000), CD44 (Abcam, 1:1000), ABCG2 (Abcam, 1:1000), ALDH1 (Abcam, 1:1000), Nanog (Abcam, 1:1000), Bmi-1 (Abcam, 1:1000), Oct-4 (Abcam, 1:1000), glyceraldehyde-3-phosphate dehydrogenase (Abcam, 1:1000), horseradish peroxidase-linked anti-rabbit IgG (CST, 1:3000), and horseradish peroxidase-linked anti-mouse IgG (CST, 1:3000). Glyceraldehyde-3-phosphate dehydrogenase was used as an internal control.

### Limiting Dilution Assay

As previously described (23), *in vitro* limiting dilution assay (LDA) was performed using ultra-low adhesion plates. After 10 days of incubation, wells without spheres were counted to analyze the efficiency of sphere formation. For *in vivo* LDA, CRC cells were serially diluted to obtain the correct number for transfer and then subcutaneously injected into the nonobese diabetic/severe combined immunodeficiency mice. Two months later, the number of tumors was recorded, and the frequency of CSCs was analyzed using the Extreme Limiting Dilution Analysis software.

### Subcutaneous Tumorigenicity

CRC cells (1 × 10^6^ cells/100 μL) were injected subcutaneously into the nude mice. Two dimensions of tumors were recorded using calipers. After 24 days, the mice were euthanized, and the tumor size was obtained using the formula (length × width^2^)/2. The heterografts were collected for immunohistochemistry (IHC) analysis.

### Liver Metastasis Assays

Using a 29-G injector, CRC cells (1 × 10^6^ cells/100 μL) were injected into the portal vein of nude mice. The mice were euthanized 8 weeks after the injection or died spontaneously. The livers were harvested, fixed in 4% paraformaldehyde, and stained with hematoxylin and eosin. The liver metastatic foci were validated and counted microscopically. The survival time was also recorded at 12 weeks as the cutoff.

### Statistical Analysis

The results were recorded as the mean ± standard error of the mean (SEM). SPSS software ver. 20.0 and GraphPad Prism 7.0 were used to performed the statistical analysis. The differences between groups were assessed using the Student’s *t*-test or analysis of variance. The Kaplan-Meier analysis was utilized to investigate the survival disparity between different groups. Cox proportional hazards models were used for univariate and multivariate analyses. The statistical significance was set at P < 0.05.

## Results

### High Expression of HIST2H2BF in CRC and Its Correlation With Poor Prognosis

**Figure 1:**
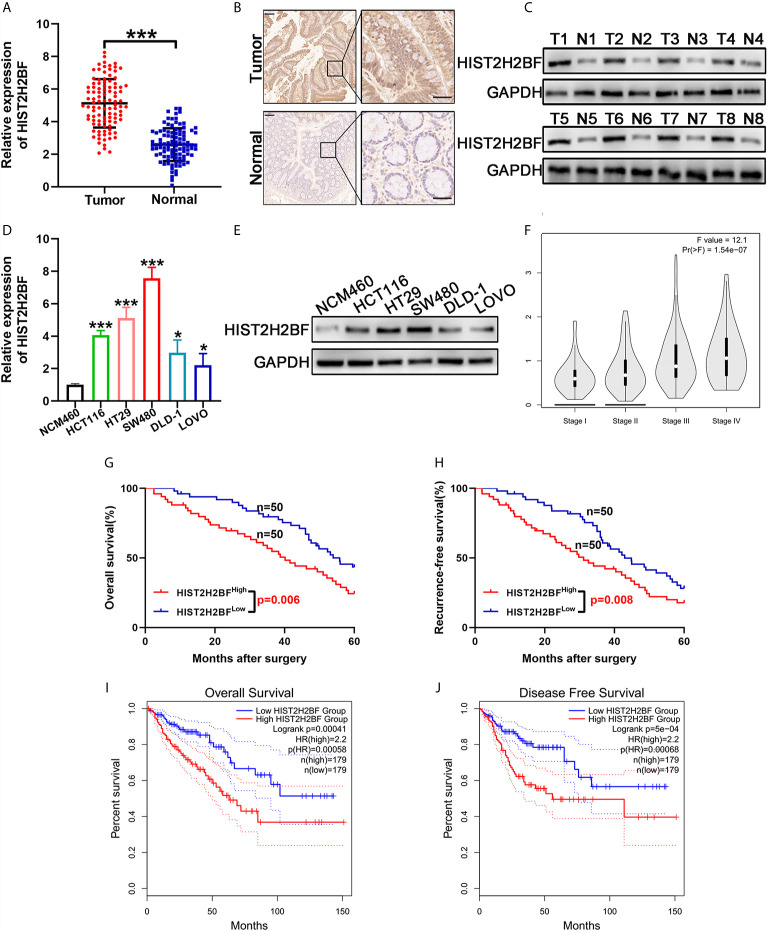
HIST2H2BF was overexpressed in CRC and relates to poor prognosis in patients with CRC. **(A)** HIST2H2BF mRNA levels were determined in 100 paired CRC and adjacent normal tissues using real-time PCR. **(B)** Representative images of HIST2H2BF expression analyzed by immunohistochemistry in paired CRC and adjacent normal tissues. Scale bars, 100 μm. **(C)** Western blotting of HIST2H2BF expression in paired CRC and adjacent normal tissues (n = 8). **(D)** The expression levels of HIST2H2BF mRNA in five CRC cell lines (LOVO, HCT116, DLD-1, HT29, and SW480) and the human normal colon epithelial cell NCM460 were detected using real-time PCR. **(E)** The expression levels of HIST2H2BF protein in CRC cell lines and NCM460. **(F)** The rxpression levels of HIST2H2BF in different stages of CRC in the GEPIA database using TCGA data. **(G, H)** The Kaplan-Meier OS **(G)** and RFS **(H)** curves of patients with CRC with high (n = 50) and low (n = 50) expressions of HIST2H2BF mRNAs in 100 paired CRC and adjacent normal tissues, respectively. **(I, J)** The Kaplan-Meier OS **(I)** and RFS **(J)** curves of patients with CRC with high (n = 179) and low (n = 179) expressions of HIST2H2BF mRNAs, respectively, in the GEPIA database using TCGA data. *P < 0.05, ***P < 0.001.

We initially compared the HIST2H2BF expression in 100 paired CRC tissues and adjacent normal tissues using reverse transcription-PCR. Results showed that HIST2H2BF was overexpressed in CRC tissues (**Figure 1A**). IHC analysis verified that HIST2H2BF protein expression was markedly higher in CRC tissues than in adjacent normal tissues (**Figure 1B**). The results of Western blotting confirmed the increased HIST2H2BF protein expression in eight randomly selected pairs of CRC tissues and adjacent normal tissues (**Figure 1C**). The HIST2H2BF expression was analyzed in five human CRC cell lines and normal human colon epithelial cells (NCM460). Consistently, both HIST2H2BF mRNA and protein expression levels were found to be elevated in CRC cell lines by reverse transcription-PCR and Western blotting, respectively (**Figures 1D, E**). The expression levels of HIST2H2BF in different stages of CRC determined using the online database GEPIA (http://gepia2.cancer-pku.cn/#analysis) showed a positive correlation with tumor stage (**Figure 1F**). The relevance analysis of HIST2H2BF expression in 100 CRC patients also revealed its positive correlation with tumor size, TNM stage, depth of invasion, and distant metastasis (**Supplemental Table 1**). The Kaplan-Meier analysis of the survival outcomes of the 100 CRC patients showed that patients with high HIST2H2BF expression had poor overall survival (OS) and recurrence-free survival (**Figures 1G, H**). This finding is consistent with the results of the analysis conducted using the online database GEPIA (**Figures 1I, J**). High HIST2H2BF expression was an independent prognostic factor for OS (hazard ratio = 1.95, 95% confidence interval: 1.23–2.87; P = 0.014) and recurrence-free survival (hazard ratio = 1.803, 95% confidence interval: 1.294–2.989; P = 0.029) in CRC patients using multivariate Cox regression analysis (**Supplemental Table 2**). These data indicate that HIST2H2BF overexpression plays a vital role in CRC progression and may predict poor clinical outcomes in CRC.

### CpG Hypomethylation Stimulating the HIST2H2BF Expression in CRC

**Figure 2:**
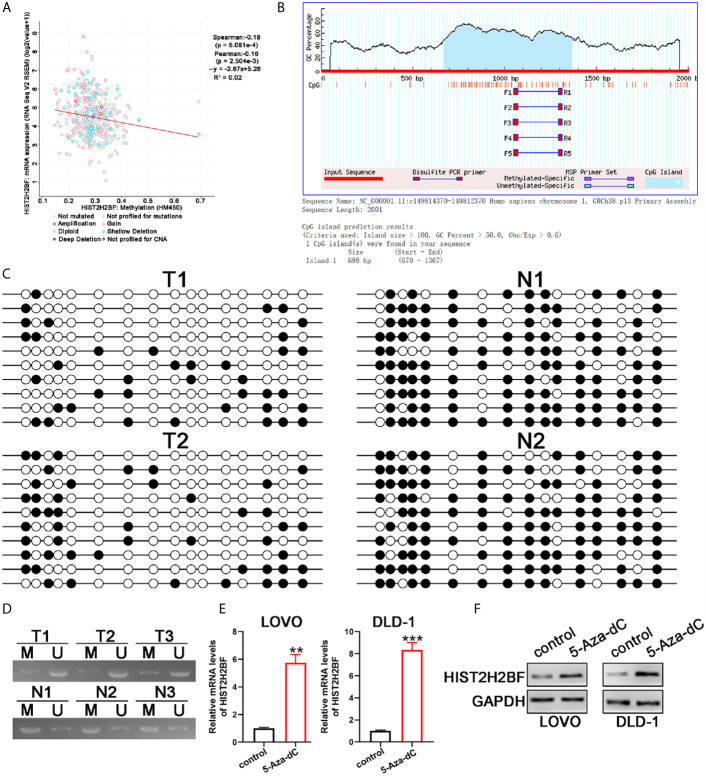
CpG hypomethylation contributes to the upregulation of HIST2H2BF in CRC. **(A)** cBioPortal data were employed to display the relevance between HIST2H2BF DNA methylation status and its mRNA expression in CRC. **(B)** MethPrimer program was employed to predict the CpG islands of the HIST2H2BF promoter and synthesis primers. **(C)** Bisulfite sequencing PCR was employed to demonstrate the methylation status of HIST2H2BF CpG islands using two paired CRC tissues and adjacent normal tissues. Open circles: unmethylated CpG sites, filled circles: methylated CpG sites. **(D)** Methylation-specific PCR was employed to determine the HIST2H2BF methylation status in three paired CRC tissues (T) and adjacent normal tissues (N). M: methylated, U: unmethylated. **(E, F)** LOVO and DLD-1 cells were treated with 5-Aza-dC. HIST2H2BF mRNA and protein expression were then detected by RT-qPCR and Western blotting, respectively. **P < 0.01, ***P < 0.001.

Next, we explored the potential mechanisms that may lead to HIST2H2BF upregulation in CRC. Data from cBioPortal database showed that, in CRC tissues, a negative correlation was observed between HIST2H2BF DNA methylation and HIST2H2BF mRNA expression (**Figure 2A**). Indeed, one typical CpG island was detected near the HIST2H2BF promoter region (**Figure 2B**). Thus, we employed bisulfite sequencing PCR analysis to determine the methylation status of the promoter region of HIST2H2BF in two matched CRC tissues and adjacent normal tissues. The bisulfite sequencing PCR results confirmed the lower CpG methylation levels in CRC tissues with higher HIST2H2BF expression (**Figure 2C**). Methylation-specific PCR analysis was then performed on the three paired CRC tissues. The methylation proportion of HIST2H2BF in CRC tissues was significantly lower than that in adjacent normal tissues (**Figure 2D**). 5‐Aza‐deoxy‐cytidine (5‐Aza‐dC) is a commonly used DNA demethylating agent. We treated the low HIST2H2BF-expressing CRC cell lines (LOVO and DLD-1) with 5-Aza-dC (5 μmol/L for 4 days). The HIST2H2BF levels were significantly elevated in LOVO and DLD-1 cells treated with 5-Aza-dC. Consistently, the HIST2H2BF protein was also elevated in LOVO and DLD-1 cells following 5-Aza-dC treatment (**Figures 2E, F**). Collectively, these data verified that the reactivation of HIST2H2BF in CRC results from the hypomethylation of CpG.

### HIST2H2BF Overexpression Markedly Enhancing the Stemness of CRC Cells

**Figure 3:**
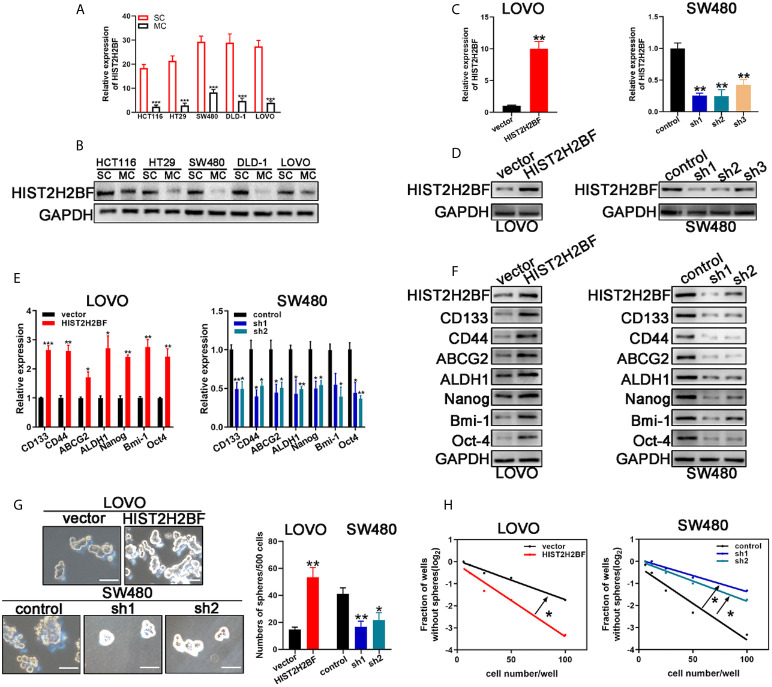
HIST2H2BF promotes stemness of CRC cells. **(A, B)** Real-time PCR and Western blotting detected HIST2H2BF levels in sphere-forming cells and monolayer cells in CRC cell lines. **(C, D)** Real-time PCR and Western blotting of HIST2H2BF expression after transfection with lentivirus HIST2H2BF or shHIST2H2BF. **(E, F)** Real-time PCR and Western blotting of the expression CSC-related biomarkers in LOVO and SW480 cells transfected with lentivirus HIST2H2BF or shHIST2H2BF. **(G)** Number of spheres in LOVO and SW480 cells infected with lentivirus HIST2H2BF or shHIST2H2BF. Scale bar, 100 μm. **(H)** LDA determined sphere formation ability following HIST2H2BF overexpression or knockdown. *P < 0.05, **P < 0.01, ***P < 0.001.

CSCs are thought to contribute to tumor initiation and development of cancers, especially in CRC. Thus, we investigated the relationship between HIST2H2BF and CSCs. Sphere-forming assays were used to separate CSCs from the CRC cell lines. In comparison with monolayer cells, sphere cells displayed higher HIST2H2BF expression at the mRNA and protein levels (**Figures 3A, B**). Furthermore, low HIST2H2BF-expressing LOVO cells were selected to overexpress HIST2H2BF by transfection with LV-HIST2H2BF. The HIST2H2BF overexpression was validated at the mRNA and protein levels (**Figures 3C, D**). In addition, high HIST2H2BF-expressing SW480 cells were selected to establish the HIST2H2BF knockdown cell lines by transfection with short hairpin RNA. We then confirmed the knockdown efficiency of three HIST2H2BF-specific short hairpin RNAs at the mRNA and protein levels (**Figures 3C, D**). Subsequent experiments were performed using sh1 and sh2, which induced the highest knockdown efficiency. HIST2H2BF overexpression increased the expression of CSC-related biomarkers (CD133, CD44, ABCG2, ALDH1, Nanog, Bmi-1, and Oct-4). By contrast, HIST2H2BF suppression decreased the CSC-related biomarker levels (**Figures 3E, F**). Spheroid formation was enhanced in HIST2H2BF-overexpressing LOVO cells and attenuated in HIST2H2BF knockdown SW480 cells (**Figure 3G**). *In vitro* limiting dilution analysis also showed that HIST2H2BF overexpression increased the sphere formation, whereas HIST2H2BF knockdown decreased the sphere formation (**Figure 3H**).

### HIST2H2BF Promoting the Proliferation, Migration, Invasion, and Drug Resistance in CRC

**Figure 4:**
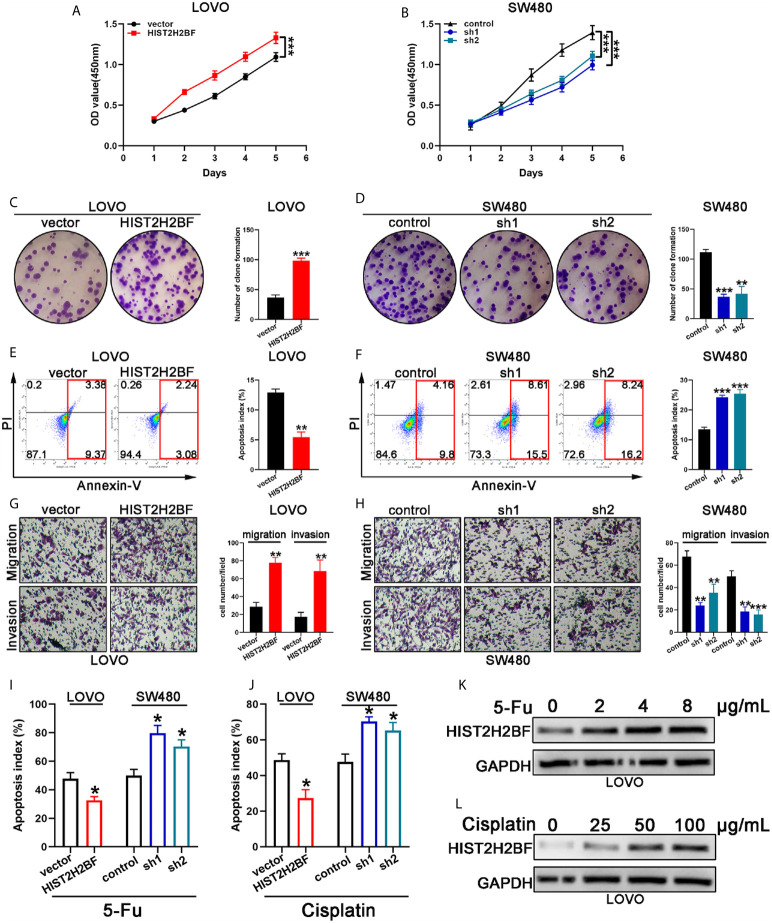
HIST2H2BF promoted proliferation, migration, invasion, and drug resistance in CRC. **(A, B)** Growth curves were recorded using CCK8 assays. **(C, D)** Colony formation assays of CRC cells; the number of colonies in each well was then counted. **(E, F)** Apoptosis of CRC cells was analyzed using a flow cytometer. **(G, H)** Migration and invasion assays of CRC cells; the number of migrated and invaded CRC cells were then counted. **(I, J)** Apoptosis of CRC cells was detected after 5-FU and cisplatin treatment. **(K, L)** Western blotting indicating HIST2H2BF expression in LOVO cells after treatment with various concentrations of 5-FU and cisplatin. *P < 0.05, **P < 0.01, ***P < 0.001.

Cell Counting Kit-8 assays demonstrated that HIST2H2BF overexpression markedly promoted the LOVO cell proliferation, while HIST2H2BF knockdown suppressed the SW480 cell proliferation (**Figures 4A, B**). Subsequently, colony formation assays confirmed the enhanced effect of increased HIST2H2BF expression on LOVO cell proliferation, while HIST2H2BF knockdown suppressed the colony formation ability in SW480 cells (**Figures 4C, D**). Apoptosis analysis revealed that HIST2H2BF overexpression in CRC cells decreased the cell apoptosis, while the opposite effect was observed in SW480 cells with HIST2H2BF knockdown (**Figures 4E, F**). Transwell assays suggested that HIST2H2BF overexpression resulted in a significant increase in the migratory and invasive ability of CRC cells. Meanwhile, HIST2H2BF downregulation decreased the migratory and invasive ability of these cells (**Figures 4G, H**). Chemoresistance is a vital trait of CSCs, and 5-FU and cisplatin are commonly used chemotherapeutic agents in the treatment of CRC. In this study, HIST2H2BF overexpression contributed to the resistance of CRC cells to apoptosis induced by 5-FU and cisplatin. However, HIST2H2BF knockdown contributed to the chemosensitivity of CRC cells to apoptosis induced by 5-FU and cisplatin (**Figures 4I, J**). In addition, following treatment with 5-FU and cisplatin, LOVO cells displayed a HIST2H2BF-enriched subpopulation (**Figures 4K, L**).

### HIST2H2BF Facilitating CRC Initiation, Progression, and Liver Metastasis *In Vivo*

**Figure 5:**
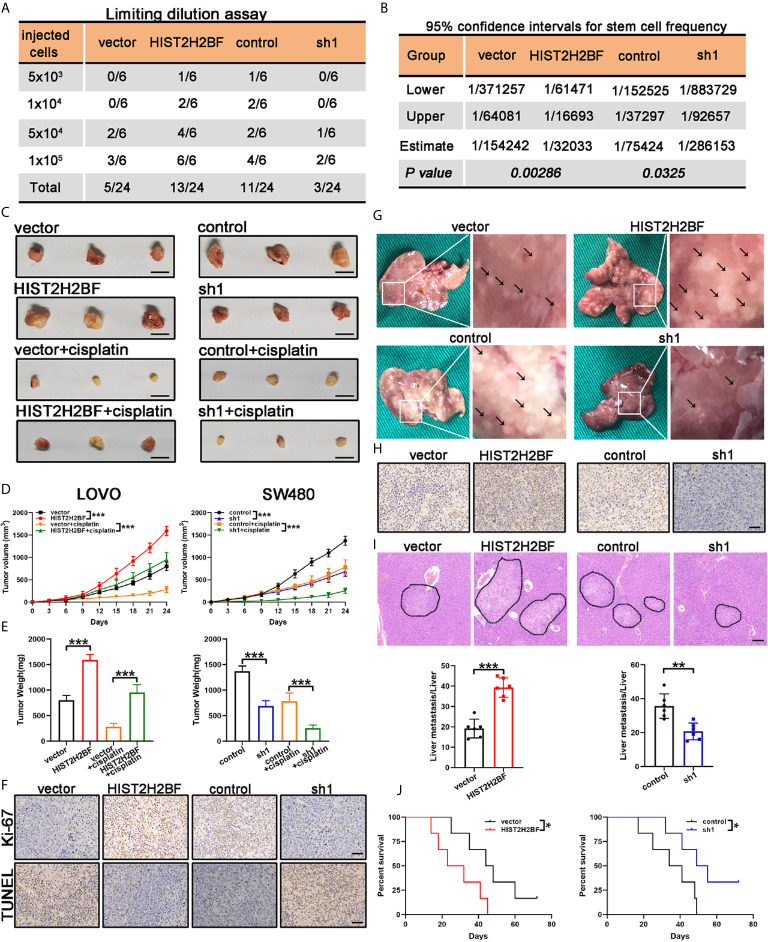
HIST2H2BF promoted tumor initiation, self-renewal, and liver metastasis in mice. **(A)** LOVO and SW480 cells transfected with lentivirus HIST2H2BF or shHIST2H2BF were injected subcutaneously in nonobese diabetic/severe combined immunodeficiency mice. The tumor-forming rates were determined 2 months post-injection. **(B)** The frequency of CSCs was obtained. **(C)** Tumors harvested from LOVO and SW480 cells, with or without cisplatin treatment. **(D)** Growth curves of LOVO and SW480 xenografts. Two-way ANOVA was employed to analyze the differences in tumor growth. **(E)** Tumor weight was weighted in LOVO and SW480 cells. **(F)** Ki-67 and TUNEL staining in the tumors harvested from LOVO and SW480 cells without cisplatin treatment. **(G)** Representative images of liver metastatic foci (marked by black arrowheads). **(H)** IHC staining for HIST2H2BF expression in the liver metastatic foci from LOVO and SW480 cells. **(I)** Hematoxylin and eosin (HE) of liver metastasis in the mouse model (upper panel). Scale bar, 200 μm. The number of liver metastatic foci were counted (lower panel). **(J)** OS of mice injected with LOVO and SW480 cells. *P < 0.05, **P < 0.01, ***P < 0.001.

We examined the impact of HIST2H2BF on CSC expansion using *in vivo* LDA. We found that HIST2H2BF overexpression in LOVO cells resulted in a higher tumor formation rate and CSC frequency. Consistently, HIST2H2BF knockdown inhibited the tumor formation rate and decreased the CSC frequency in CRC cells (**Figures 5A, B**). Moreover, HIST2H2BF overexpression in CRC cells increased the tumor growth, volume, and weight (**Figures 5C–E**). IHC staining showed decreased apoptosis and increased proliferation in the HIST2H2BF-overexpressed xenografts, as revealed by Ki-67 and TUNEL staining (**Figure 5F**). Moreover, compared with the controls, the combination of HIST2H2BF overexpression and cisplatin treatment promoted tumor growth by as much as 152%, indicating the promotive role of HIST2H2BF in CSC propagation and development (**Figures 5C–E**). As expected, HIST2H2BF knockdown decreased the tumor growth, volume, and weight (**Figures 5C–E**). Meanwhile, Ki-67 and TUNEL staining also exhibited decreased proliferation and increased apoptosis rate in SW480-sh1 xenografts, respectively (**Figure 5F**). As expected, HIST2H2BF knockdown suppressed the tumor growth following cisplatin treatment in comparison with the effects observed in the control group (**Figures 5C–E**). Liver metastasis models were established through adoptive CRC cell transfer into the nude mice *via* the portal vein. The biological effect of HIST2H2BF on liver metastasis was further investigated using LOVO and SW480 cells transfected with HIST2H2BF-overexpressed and knockdown vectors, respectively (12 mice/group) (**Figure 5G**). IHC staining confirmed that the protein levels of HIST2H2BF were consistent with those observed *in vitro* (**Figure 5H**). There were significantly more liver metastases in the LOVO-HIST2H2BF group, while the opposite result was observed in the HIST2H2BF knockdown group (**Figure 5G**). HE staining of liver metastasis was further performed to verify the metastatic nodules (**Figure 5I**). Compared with the corresponding control mice, HIST2H2BF overexpression and knockdown mice also displayed shorter and longer OS, respectively (**Figure 5J**). Collectively, these data suggest that HIST2H2BF plays a significant role in facilitating CRC progression and liver metastasis.

### HIST2H2BF Activating the Notch Signaling to Promote Stemness and Malignancy of CRC

**Figure 6:**
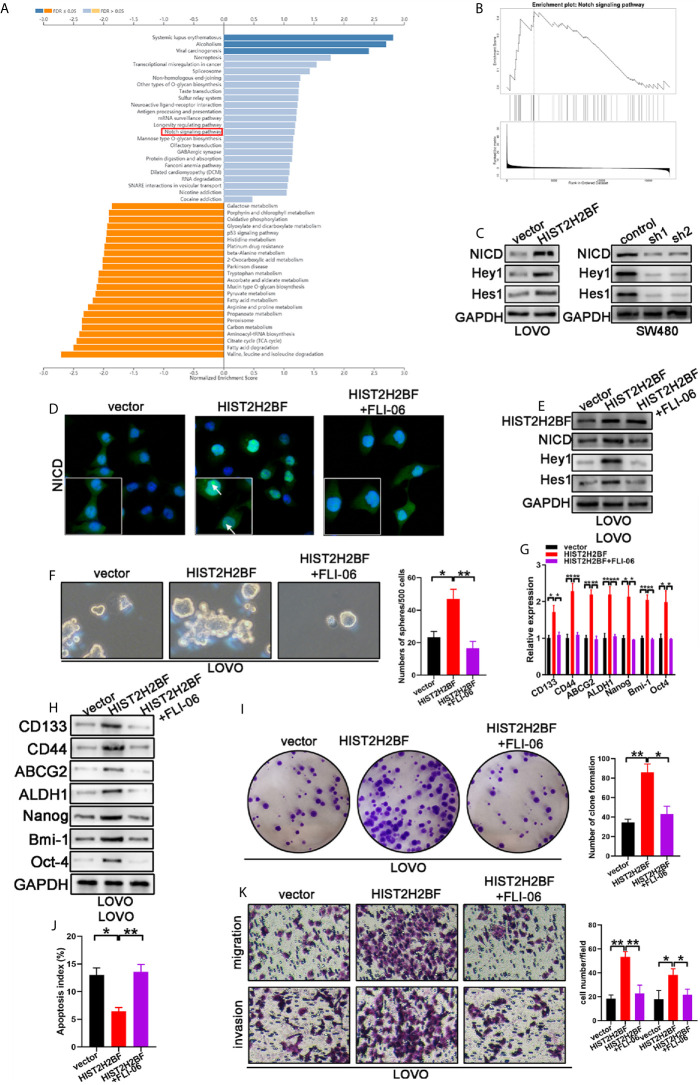
HIST2H2BF activates Notch signaling to promote the development of stem cell-like and cancer properties in CRC. **(A)** Kyoto Encyclopedia of Genes and Genomes (KEGG) analysis was performed *via* LinkedOmics. **(B)** Notch signaling is regulated by HIST2H2BF. **(C)** Western blotting indicated NICD, Hey1, and Hes1 expression in LOVO and SW480 cells infected with lentivirus HIST2H2BF or shHIST2H2BF. **(D)** Fluorescence microscopy analysis for NICD in LOVO cells. Arrowheads indicate the expression of NICD in the nucleus. Green, NICD; blue, nucleus. **(E)** Following FLI-06 treatment, Western blotting indicated NICD, Hey1, and Hes1 expression in LOVO cells infected with lentivirus HIST2H2BF. **(F)** Following FLI-06 treatment, the spheres were counted in LOVO cells infected with lentivirus HIST2H2BF. **(G)** Following FLI-06 treatment, the expression CSC-related biomarkers were detected in LOVO cells infected with lentivirus HIST2H2BF using real-time PCR. **(H)** Following FLI-06 treatment, the expression CSC-related biomarkers were detected in LOVO cells infected with lentivirus HIST2H2BF using Western blotting. **(I)** Following FLI-06 treatment, colony formation assays were carried out in LOVO cells infected with lentivirus HIST2H2BF. **(J)** Following FLI-06 treatment, apoptosis assays were carried out in LOVO cells infected with lentivirus HIST2H2BF. **(K)** Following FLI-06 treatment, migration and invasion assays were carried out in LOVO cells infected with lentivirus HIST2H2BF. *P < 0.05, **P < 0.01, ***P < 0.001.

Investigation of the downstream signaling of HIST2H2BF is crucial to uncover the mechanisms underlying the HIST2H2BF regulation of stemness and malignancy of CRC. Kyoto Encyclopedia of Genes and Genomes (KEGG) analysis was then performed using LinkedOmics (http://www.linkedomics.org/login.php) (**Figure 6A**). The data revealed that Notch signaling was regulated by HIST2H2BF (**Figure 6B**). Previous studies have shed light on the significant role of Notch signaling in promoting stemness and malignancy. As shown in **Figure 6C**, HIST2H2BF overexpression contributed to the release of the Notch intracellular domain (NICD) and upregulated the downstream target genes, including Hes-1 and Hey-1 in LOVO cells. By contrast, HIST2H2BF knockdown significantly suppressed the Notch signaling in SW480 cells. Consistently, the cellular immunofluorescence assay indicated a similar effect. As shown in **Figure 6D**, HIST2H2BF overexpression enhanced the fluorescence intensity of NICD in the nucleus. These results indicated that HIST2H2BF promotes the expression of NICD, the activated form of Notch1, and this effect may further promote downstream gene transcription. To confirm that HIST2H2BF activates Notch signaling to promote stemness and malignancy in CRC, we treated the LOVO cells with FLI-06 (Notch pathway suppressor) for 2 days (24). Immunofluorescence assay and Western blotting verified the FLI-06-induced inactivation of Notch signaling (**Figures 6D, E**). Meanwhile, the FLI-06-induced inhibition of Notch signaling reversed these stem cell-like properties, based on the results of spheroid formation assays and CSC-related biomarker levels (**Figures 6F–H**). In addition, the enhanced proliferative, migratory, and invasive ability of HIST2H2BF overexpression in LOVO cells was rescued by treatment with FLI-06 (**Figures 6I–K**). These results suggest that HIST2H2BF activates Notch signaling to promote stemness and malignancy in CRC.

## Discussion

Extensive evidence indicates that CRC is attributed to various factors, including genetic, molecular, and epigenetic alterations (25, 26). An understanding of the underlying mechanisms and factors that facilitate the development and progression of CRC can aid in the exploration of specific therapeutic targets to improve the standard treatments. Our results indicated an obvious overexpression of HIST2H2BF in CRC tissues and cell lines. Moreover, high HIST2H2BF expression is an independent prognostic biomarker for OS and recurrence-free survival in patients with CRC. Both *in vitro* and *in vivo* assays revealed that HIST2H2BF promotes malignant tumor behaviors in CRC.

DNA methylation deregulation contributes markedly to tumor progression and acts as a vital marker to predict the response to therapy and prognosis in tumors (27, 28). In our study, we examined the methylation levels of the HIST2H2BF promoter with methylation-specific PCR and bisulfite sequencing PCR in CRC tissues and adjacent normal tissues. Our results showed a lower methylation level of the HIST2H2BF promoter in CRC tissues, suggesting that promoter hypomethylation might contribute to HIST2H2BF transcription. As expected, HIST2H2BF was also significantly upregulated following 5’-Aza-dC treatment. To our knowledge, this is the first study to report the association of hypomethylation of the HIST2H2BF promoter with transcriptional upregulation of HIST2H2BF in CRC.

CSCs account for tumor initiation, development, progression, and metastasis (29, 30). CSCs are also attributed to tumor relapse, resistance to chemotherapy, and radiation therapy, which are common clinical events (31). Thus, therapies targeting cells may be a potential strategy for cancer therapy. In our study, we found that HIST2H2BF was highly expressed in CSCs. HIST2H2BF overexpression *in vitro* significantly increased the sphere formation and gene expression levels related to stemness. A series of *in vivo* experiments also indicated that HIST2H2BF contributes to tumor initiation and liver metastasis in CRC.

The Notch pathway, which is an extremely conserved pathway, accounts for the direct cell-to-cell interactions in multicellular organisms (32, 33). The normal state of the Notch pathway is important for maintaining cell proliferation, apoptosis, development, and differentiation (34, 35). Growing evidence suggests that the activation of the Notch pathway plays an oncogenic role in CRC tumorigenesis (36). The activation of Notch1 upregulated the expression of the downstream targets, Hes-1, and Hey-1 in CRC cells (37). Recent research also claimed that the activity of the Notch pathway in the early stage of CRC is comparatively elevated compared with that in the advanced stage. Notch signaling mainly promotes CRC development and progression by regulating the cell cycle and apoptosis (38). Moreover, accumulating evidence has confirmed that Notch signaling plays a vital role in the development of cancer stem cell-like properties (39). In our study, we found that HIST2H2BF activates Notch signaling to promote stemness and malignancy in CRC. FLI-06, a Notch pathway suppressor, reversed the stemness and malignancy. However, given the complexity of disruption of the signaling pathways in CRC such as the JNK signaling pathway and Wnt signaling pathway (40–42), it remains unclear whether HIST2H2BF also affects other pathways to promote stemness and malignancy, which needs further investigation.

In conclusion, we found that hypomethylation-induced HIST2H2BF upregulation enhances the CSC phenotype, malignancy, and liver metastasis through the activation of Notch signaling in CRC. This provides a new perspective on the mechanism by which the stem cell features of CRC cells are maintained and highlights potential novel therapeutic targets for CRC.

## Data Availability Statement

The datasets presented in this study can be found in online repositories. The names of the repository/repositories and accession number(s) can be found in the article/**Supplementary Material**.

## Ethics Statement

The studies involving human participants were approved by the Research Ethics Committee of The First Affiliated Hospital of Nanjing Medical University. The patients provided their written informed consent. The animal study was approved by the Animal Research Committee of The First Affiliated Hospital of Nanjing Medical University.

## Author Contributions

ZF and YM conceived the project and designed the research. LQ carried out the experiments *in vitro*. XY were responsible for the experiments *in vivo*. JW drafted the manuscript. CH performed the data analysis. ZF and LQ were responsible for quality control. All authors contributed to the article and approved the submitted version.

## Funding

This work was supported by the National Natural Science Foundation of China (81470881), the Natural Science Foundation of Jiangsu Province (WSW‐025), the Key Medical Subjects of Jiangsu Province (H201116), and Youth Science and Technology Project of Lianyungang Health Commission (QN1809).

## Conflict of Interest

The authors declare that the research was conducted in the absence of any commercial or financial relationships that could be construed as a potential conflict of interest.

## Publisher’s Note

All claims expressed in this article are solely those of the authors and do not necessarily represent those of their affiliated organizations, or those of the publisher, the editors and the reviewers. Any product that may be evaluated in this article, or claim that may be made by its manufacturer, is not guaranteed or endorsed by the publisher.
