# Supplementary material for: HIST2H2BF Potentiates the Propagation of Cancer Stem Cells via Notch Signaling to Promote Malignancy and Liver Metastasis in Colorectal Carcinoma
Source: Front Oncol. 2021 Aug 12;11:677646. doi: 10.3389/fonc.2021.677646 (PMC8406628; doi:10.3389/fonc.2021.677646)
Supplement: Supplementary file 1 [file Table_1.doc]

| **Supplemental Table1** Expression of HIST2H2BF in CRC according to patients’ clinicopathological characteristics | | | | |
| --- | --- | --- | --- | --- |
| Characteristics | Number(%) | HIST2H2BF expression | | P-value |
|  |  | High group | Low group |  |
| **Age(years)** |  |  |  |  |
| <60 | 39(48.0%) | 19 | 20 | 0.838 |
| ≥60 | 61(52.0%) | 31 | 30 |  |
| **Gender** |  |  |  |  |
| Male | 71(64.0%) | 38 | 33 | 0.271 |
| Female | 29(36.0%) | 12 | 17 |  |
| **Tumor size** |  |  |  |  |
| <5 | 57(51.0%) | 16 | 27 | **0.026*** |
| ≥5 | 43(49.0%) | 34 | 23 |  |
| **TNM stage** |  |  |  |  |
| I/II | 44(52.0%) | 16 | 28 | **0.016*** |
| III/IV | 56(48.0%) | 34 | 22 |  |
| **Depth of invasion** |  |  |  |  |
| T1+T2 | 48(45.0%) | 17 | 31 | **0.005**** |
| T3+T4 | 52(55.0%) | 33 | 19 |  |
| **Distant metastasis** |  |  |  |  |
| Yes | 25(51.0%) | 18 | 7 | **0.011*** |
| No | 75(49.0%) | 32 | 43 |  |
| **CEA (ng/ml)** |  |  |  |  |
| ≥5 | 86(36.0%) | 40 | 46 | 0.084 |
| <5 | 14(64.0%) | 10 | 6 |  |
| **Lymph node metastasis** |  |  |  |  |
| Yes | 32(36.0%) | 15 | 17 | 0.668 |
| No | 68(64.0%) | 35 | 33 |  |
| **Tumor location** |  |  |  |  |
| Colon | 68(45.0%) | 36 | 32 | 0.391 |
| Rectum | 32(55.0%) | 14 | 18 |  |

| *****p＜0.05 , ******P<0.01 , **^#^**P<0.001 |
| --- |
|  |
