# Supplementary material for: HIST2H2BF Potentiates the Propagation of Cancer Stem Cells via Notch Signaling to Promote Malignancy and Liver Metastasis in Colorectal Carcinoma
Source: Front Oncol. 2021 Aug 12;11:677646. doi: 10.3389/fonc.2021.677646 (PMC8406628; doi:10.3389/fonc.2021.677646)
Supplement: Supplementary file 2 [file Table_2.doc]

Table 2 Univariate and multivariate analysis of factors associated with overall survival and recurrence-free survival of 100 CRC patients

|  | |  | Overall survival Recurrence-free survival | | | | | | |
| --- | --- | --- | --- | --- | --- | --- | --- | --- | --- |
| Clinicopathologic Parameters |  | Univariate | Multivariate analysis | | | Univariate | Multivariate analysis | | |
|  | | *P* value | HR | 95% CI | *P* value | *P* value | HR | 95% CI | *P* value |
| **Age (>50 years vs ≤50 years)**  **Gender (female vs male)**  **Tumor size (≥5cm vs <5cm)**  **TNM stage (III/IV vs I/II)**  **Depth of invasion (T3+T4 vs T1+T2)**  **Distant metastasis (Yes vs No)**  **CEA (≥5ng/ml vs <5ng/ml)**  **Lymph node metastasis (Yes vs No)**  **Tumor location (Colon vs Rectum)**  **HIST2H2BF expression (High vs Low)** | | 0.610 |  |  |  | 0.899 |  |  |  |
|  |  | 0.354 |  |  |  | 0.320 |  |  |  |
|  |  | 0.095 |  |  |  | 0.119 |  |  | |
|  |  | **0.016^*^** | NI |  |  | **0.019^*^** | NI |  |  |
|  |  | **0.008^**^** | NI |  |  | 0.229 |  | | |
|  |  | **<0.001^***^** | 2.99 | 1.65-3.97 | **0.003^**^** | **<0.001^***^** | 3.002 | 1.902-4.012 | **0.002^*^** |
|  |  | 0.278 | NI |  |  | 0.516 |  | | |
|  |  | **0.003^**^**  0.778  **0.006^**^** | 1.33  1.95 | 1.13-3.04  1.23-2.87 | **0.046^*^**  **0.014^*^** | **0.007^**^**  0.166  **0.008^**^** | 1.791  1.803 | 1.193-3.286  1.294-2.989 | **0.034^*^**  **0.029^*^** |

*p < 0.05,**p < 0.01,***p < 0.001

^a^Using median HIST2H2BF values as cutoff

HR, hazard ratio; CI, confidence interval; TNM, tumor-node-metastasis; NI, not included.

The bold number means statistically significant.
